# Activation of the *NF‐ĸB* Signaling Pathway by *Lactobacillus plantarum* and *Lactobacillus casei* Through Modulation of *TLR4* and *DC‐SIGN* Genes in an Oral Squamous Cell Carcinoma Rat Model

**DOI:** 10.1002/cnr2.70505

**Published:** 2026-05-15

**Authors:** Vahideh Faghanizadeh, Malihe Naderi, Nazila Arbab Soleimani, Ayyoob Khosravi, Mohammad Mahdi Forghanifard

**Affiliations:** ^1^ Department of Microbiology Damghan Branch, Islamic Azad University Damghan Iran; ^2^ Department of Tropical Viral Vaccine Development Institute of Tropical Medicine, Nagasaki University Nagasaki Japan; ^3^ Department of Microbiology and Microbial Biotechnology, Faculty of Life Sciences and Biotechnology Shahid Beheshti University Tehran Iran; ^4^ Stem Cell Research Center, Biomedical Research Institute, Golestan University of Medical Sciences Gorgan Iran; ^5^ Department of Molecular Medicine, Faculty of Advanced Medical Technologies Golestan University of Medical Sciences Gorgan Iran; ^6^ Departments of Biology, Damghan Branch Islamic Azad University Damghan Iran

**Keywords:** *BCL2*, *CLR*, *Lactobacillus casei*, *Lactobacillus plantarum*, *NF‐ĸB*, OSCC, *PRRs*, *TLRs*

## Abstract

**Background:**

The composition of the oral and intestinal microbiota is believed to contribute to the development of oral cancer (OC). The chronic activation of pattern recognition receptors (PRRs) has been linked to the aggressive nature and poor prognosis of various cancers. Therefore, targeting PRRs has emerged as a promising strategy for cancer prevention.

**Aims:**

To investigate the effects of *Lactobacillus plantarum* (*LP*) and *Lactobacillus casei* (*LC*) on downstream signaling pathways of *CLRs* in a rat model of oral squamous cell carcinoma (OSCC).

**Methods and Results:**

In this study, we divided 35 rats into five groups: healthy control, cancer control, treated with live LP, treated with killed *LP*, and treated with live *LC*. Our investigation focused on gene expression of *TLR4*, *DC‐SIGN*, *NF‐κB*, and *BCL2* in healthy and cancer control groups. We established a 4NQO‐induced rat tongue carcinogenesis model and administered probiotic lactobacilli as a treatment. Subsequently, we re‐evaluated the expression of the *TLR4*, *DC‐SIGN*, *NF‐κB*, and *BCL2* genes. The antibacterial activity of both LP and LC was confirmed using the disk diffusion antibiogram method. Histopathological analysis confirmed the onset of OSCC. Expression of *the NF‐κB*, *BCL2*, *DC‐SIGN*, and *TLR4* genes was significantly increased in the 4NQO treatment group compared with the control group. Our findings indicate that live and killed *LP* and live *LC* significantly reduced the expression of the *NF‐κB*, *BCL2*, *DC‐SIGN*, and *TLR4* genes in cancerous rats compared with healthy rats.

**Conclusion:**

Our findings suggest that the administration of *LP* and *LC* probiotics may be a promising therapeutic approach for OSCC. The *LP* and *LC* can activate the *NF‐ĸB* signaling cascade by modulating the expression of *TLR4* and *DC‐SIGN* genes. However, further research is required to fully elucidate the mechanisms underlying these effects and determine the optimal dosage and duration of treatment.

## 1 Introduction

Head and neck cancer is characterized by malignant tumors that originate from the epithelial cells lining the upper aerodigestive tract and can potentially metastasize to other parts of the body [1]. Oral squamous cell carcinoma (OSCC) is a type of head and neck cancer that arises from the cells in the oral cavity, including the tongue, gums, and cheeks [2]. According to the WHO, approximately 300 000 new cases of oral cancer (OC) are diagnosed each year, and more than 90% of them are OSCC [3]. In Iran, OSCC is the fourth most common cancer, and its incidence has been increasing in recent years [4]. Like other cancers, OSCC is influenced by a range of factors, including lifestyle, genetics, and environment [5]. Emerging evidence suggests that dietary factors [6], such as iron deficiency [7], and infectious agents, such as human papillomavirus (HPV) [8], may also play a role in the development of OSCC [9]. There have been only a few studies on the microbial composition of individuals with OSCC. Recent research has identified significant differences between the microbial flora of healthy individuals and those with OSCC [10, 11]. It is therefore essential to conduct further research to enhance our understanding of the biological mechanisms underlying OSCC and to identify new biomarkers and predictors that can improve treatment outcomes.

Probiotics are beneficial microorganisms found in fermented foods and supplements that may have anti‐cancer properties and enhance the immune system's ability to fight cancer [12]. Some of the most common probiotic microorganisms include *Lactobacillus* [13], *Bifidobacterium* [14], and *Saccharomyces cerevisiae* subspecies *Boulardii* [15]. Lactic acid bacteria are particularly important among probiotics, with *Lactobacillus* and *Bifidobacterium* being natural components of the digestive system and fermented foods [16]. There is a growing interest in exploring the potential use of probiotics, which can support healthy microbiota, as a complementary therapy in cancer treatment [17].

*Lactobacillus casei* ( *L. casei* ; *LC*) has been used as a probiotic in the food industry and may have anticancer properties [18, 19, 20]. However, studies have shown that *Lactobacillus plantarum* (*L. plantarum; LP*) can improve oral health by reducing inflammation and promoting the growth of beneficial bacteria in the oral microbiome [21, 22]. Some studies have also suggested that *LP* has anti‐cancer properties and could inhibit the growth and spread of OSCC cells [23]. The precise immunological factors that contribute to *LC*‐ and *LP*‐induced cancer cell death are not yet clear; the bacterium's pathogen‐associated molecular patterns (PAMPs) may activate innate immunity through pattern recognition receptors (PRRs).

PRRs are proteins used by cells of the innate immune system, facilitating the uptake of pathogens by cells and mediating antimicrobial and pro‐inflammatory effects [24, 25]. Known pattern recognition receptors include Toll‐like receptors (*TLRs*) [26], Nod‐like receptors [27], RIG‐I‐like receptors [28], C‐type lectin receptors (*CLRs*) [29], and cyclic GMP‐AMP synthase [30]. *TLR4* is a central receptor that plays a critical role in regulating inflammation and immune responses [31]. The *NF‐κB* transcription factor controls the transcription of target genes related to cell survival, proliferation, apoptosis, invasion, and metastasis [32]. *CLRs* are also crucial in recognizing pathogens and regulating immune responses, with *DC‐SIGN* being a specific type that recognizes high‐mannose N‐glycans on viruses, bacteria, and fungi. Recent studies have shown that *DC‐SIGN* can initiate innate immunity by regulating *TLRs*, in addition to its role as an adhesion molecule [22]. Accordingly, the study aimed to understand the effects of *LP* (killed or live) and *LC* on the downstream signaling pathways of *CLRs* in a rat model of OSCC. Accordingly, this study investigates the effects of *LP* and *LC* on OSCC using a 4NQO‐induced rat model. It examines their role in modulating immune‐related genes (*TLR4, DC‐SIGN, NF‐κB*, and *BCL2*) and compares live vs. heat‐killed bacteria to determine their therapeutic potential. Given OSCC's rising incidence and limited treatment options, this research highlights probiotics as a promising alternative for future cancer therapy.

## 2 Materials and Methods

### 2.1 Microbial Strains, Reagents, and Culture Conditions

From April to March 2021, an experimental study was conducted at the Stem Cell Research Center, Faculty of Advanced Medical Technologies, Golestan University of Medical Sciences (GoUMS), Iran. The microorganisms *L. casei* (PTTC:1608) and *L. plantarum* (ATTC:8014) were obtained from the Persian Type Culture Collection (PTCC) and used to treat OC in rats. For culturing the microorganisms, MRS agar (HiMedia, Mumbai) was used as the standard medium. The probiotic properties of *LP* and *LC* were confirmed through a series of biochemical tests. The suspension of lactobacilli for the treatment study contained approximately 10^8^ CFU/mL and was prepared in 0.85% saline solution. To assess the characteristics of lactobacilli grown on MRS agar, various tests were performed, including microscopic examination (Gram staining), measurement of catalase activity, growth at different NaCl concentrations, and fermentation of carbohydrates. Additionally, assessments were made of bile and acid resistance, as well as antibiotic sensitivities [33].

### 2.2 Animals' Homing and Treatment Groups

**FIGURE 1:**
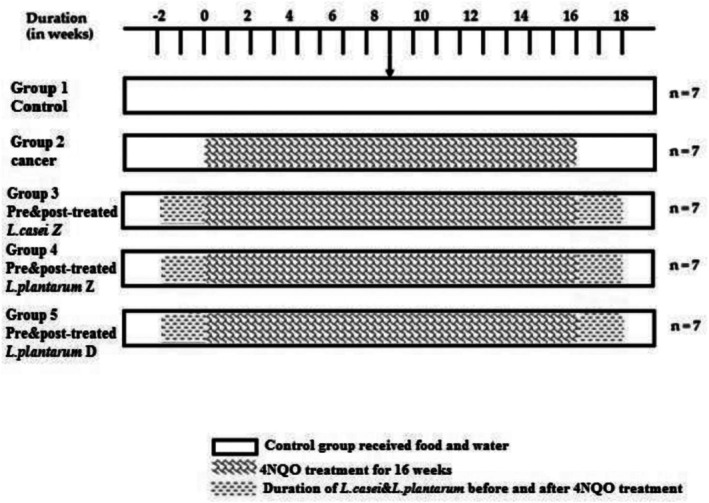
The schematic representation of 4NQO treatment and *Lactobacillus casei* and *Lactobacillus plantarum* gavage. *L. casei* Z: Live *L. casei* ; *L. plantarum* D: Dead *L. plantarum* .

A total of 35 male Wistar rats weighing an average of 300 g were obtained from the Pasteur Institute of Amol, Mazandaran, Iran. The rats were quarantined for at least 1 week and underwent a physical examination to ensure their suitability for the study. They were housed in polycarbonate shoebox cages with hardwood bedding, seven rats per cage, in a windowless room with a 12/12 h light/dark cycle. Standard maintenance conditions were 18°C–22°C, 20%–25% humidity. The rats had free access to Purina 5001 laboratory diet and tap water, which were replaced twice weekly. The rats were divided into five groups: Group 1 (*n* = 7) served as the control group and received only food and tap water; Group 2 (*n* = 7) was treated with 4NQO to induce cancer; Group 3 (*n* = 7) was gavaged with live *LC* for 2 weeks after carcinogenesis; Group 4 (*n* = 7) was treated with live *LP*; and Group 5 (*n* = 7) was treated with killed (heated) *LP* (Figure 1). All animal procedures were approved by the Research Ethics Committee of Islamic Azad University, Damghan, Iran (code number: 1401.002).

### 2.3 Generating the 4NQO‐Induced Animal Model of OSCC

To induce OSCC in rats, 4‐Nitroquinoline 1‐oxide (4NQO) from Sigma‐Aldrich (St. Louis, MO, USA) was orally administered. The rats were given anesthesia through peritoneal administration of xylazine‐ketamine, and their tongues were rubbed once with a number 3 camel hair brush before being treated with the 4NQO solution. The rats were not allowed to drink water for the first hour after receiving the treatment. A solution containing 20 ppm of this substance was dissolved in propylene glycol and administered to rats three times a week for 16 weeks [34]. A fresh solution was used each time, and the rats were weighed weekly while their tongues were checked for any changes in appearance.

### 2.4 Obtaining Tissue Samples and Pathological Examinations

After 16 weeks, the rats were anesthetized and then euthanized using a CO_2_ box. Tissue samples were obtained using a punch biopsy. The palatal mucosa and tongue were cut in half, and any visible abnormalities were measured and frozen in liquid nitrogen before being stored at −70°C. The palatal mucosa was cut in a vertical plane, while the tongues were cut horizontally. The frozen tissues were sliced into 6–8 μm‐thick sections, fixed in 10% formalin, and stained with hematoxylin and eosin (H&E).

### 2.5 Real‐Time PCR Gene Expression Assay

The study utilized real‐time PCR to evaluate the expression of *TLR4*, *NF‐κB*, *DC‐SIGN*, and *BCL2* in blood samples from rats before and after carcinogenesis. RNA was extracted from 0.5–1 mL of whole blood using the RNeasy KMini kit (*Yekta Tajhiz Azma, Tehran, Iran*), and its quantity and quality were assessed by optical density measurements and gel electrophoresis. cDNA was synthesized using the QuantiTect Reverse Transcription Kit (*Yekta Tajhiz Azma*), and RT‐PCR was performed using the QuantiTect SYBR Green PCR Kit (*Yekta Tajhiz Azma*). The SYBR Green technique was used for comparative real‐time PCR in triplicate on the Stratagene Mx3000P. The Oligo7 program generated the primer sets used (Table 1). The real‐time PCR temperature profile was as follows: 95°C (10 min), 39 cycles at 95°C (15 s), 60°C (20 s), and 72°C (20 s). The 2^−ΔΔCt^ method was used to assess gene expression fold changes, with actin as the housekeeping gene [28, 29]. The list of primers can be found in Table 1.

**TABLE 1:** List of primers.

Primer	Orientation	5′ to 3′ sequence	Amplicon size (bp)
*BCL2*	F	GGGTCATGTGTGTGGAGAG	181
	R	AGCCAGGAGAAATCAAACAG	
*NF‐ĸB*	F	TTCCCCTGTACGATAGTCGG	77
	R	GTGCTAGAAGCTGGAGGATG	
*TLR4*	F	GCTTCTCCAATTTCTCACAAC	201
	R	AGGTCATTTTTGTCTCCACAG	
*DC‐SIGN*	F	GGCGGCCCTGTACTTTTTG	502
	R	AAGGCAGCCAAGCAAGGAC	
*GAPDH*	F	AGCTCATTTCCTGGTATGACA	125
	R	TTGCTCTCAGTATCCTTGC	

### 2.6 Statistical Analysis

The data analysis aimed to compare the differences in the expression of the *TLR‐4*, *NF‐κB*, and *DC‐SIGN* genes between the control group and the *LC*‐ or *LP*‐treated groups. Statistical significance was assessed using *p* values < 0.05. Gene expression levels were determined using two‐way ANOVA and a Tukey post hoc test.

## 3 Results

### 3.1 Antibacterial Activity of Lactobacilli

**FIGURE 2:**
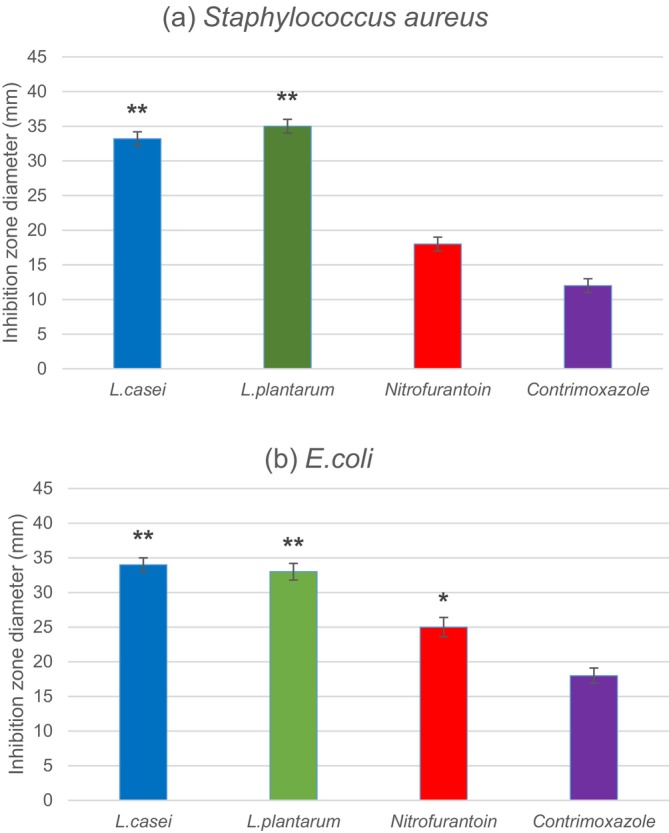
The average diameter of the growth inhibition of *Staphylococcus aureus* (a) and *Escherichia coli* (b) in the presence of *Lactobacillus casei* *and Lactobacillus plantarum*. It was found that the complete culture of *Lactobacillus casei* and *Lactobacillus plantarum*, with an average inhibition of 33–35 mm, has a strong ability to inhibit the pathogenic bacteria *Escherichia coli* (PTCC 1396) and *Staphylococcus aureus* (ATCC 25923). *p value < 0.05, **p value < 0.01. Nitrofurantoin and cotrimoxazole antibiotics were used as the control group for comparison.

The modified two‐layer culture method was used to evaluate the antibacterial activity of the complete culture of *LC* and *LP* against pathogenic bacteria, including *Escherichia coli* (PTCC: 1396) and *Staphylococcus aureus* (ATCC: 25923). The results showed that the average growth‐inhibition diameter of *LC* against *E. coli* and *S. aureus* was 34 and 33.2 mm, respectively. Similarly, *LP* exhibited an antibacterial effect of 33 and 35 mm against *E. coli* and *S. aureus* , respectively (Figure 2). Nitrofurantoin and cotrimoxazole were used as controls for comparison.

### 3.2 Clinical and Pathologic Findings

**FIGURE 3:**
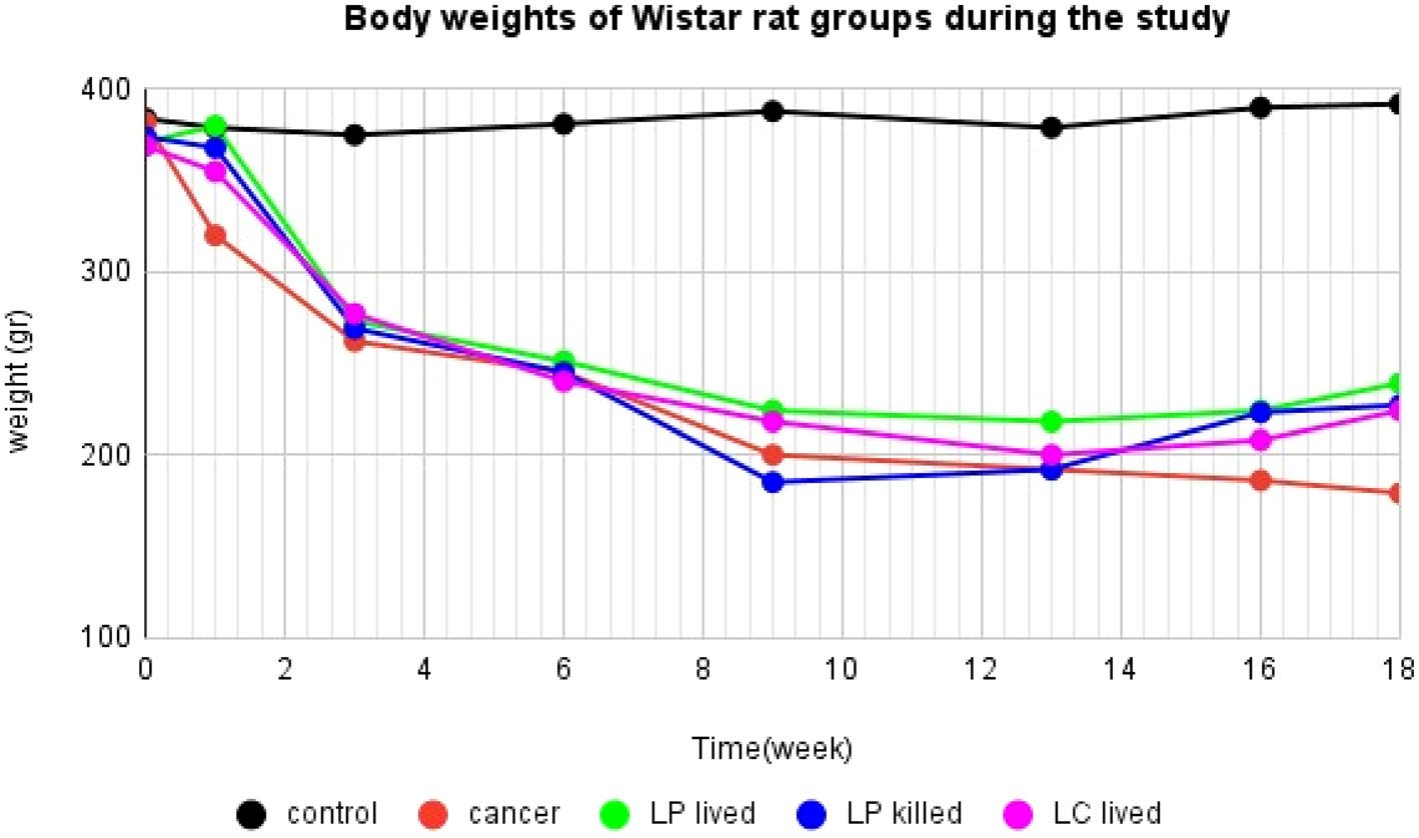
Body weights of groups of Wistar rats at different times during the study. The results show that rat body weight decreased over time with the 4NQO solution. *L. casei* Z: Live *L. casei* ; *L. plantarum* D: Dead *L. plantarum* .

**FIGURE 4:**
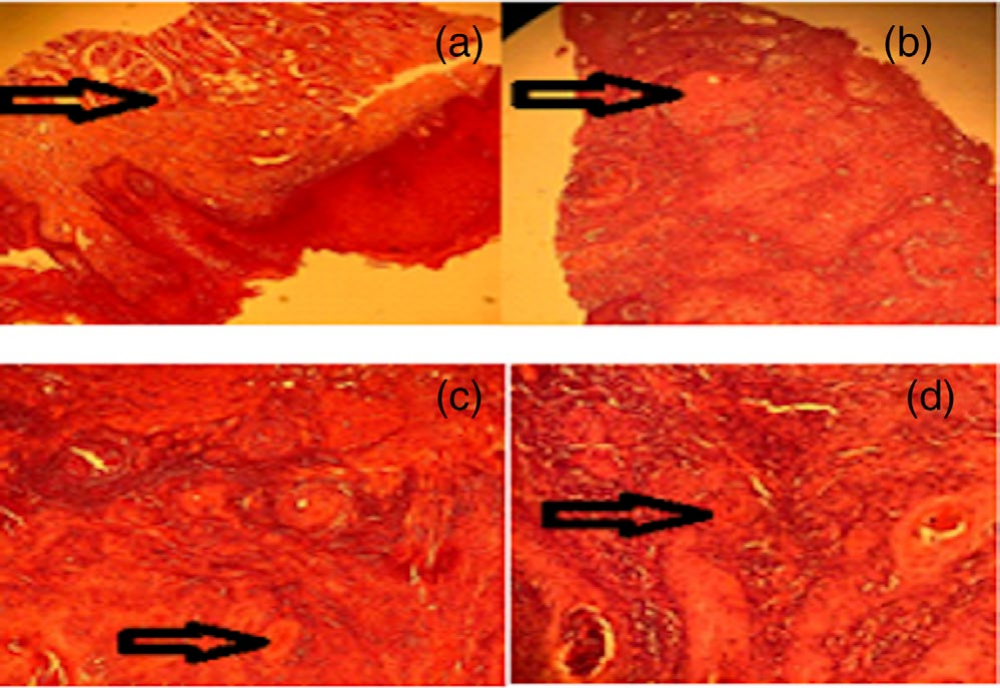
Pathological findings of the mouse tongue mass before and after administration of 4NQO. (a) Microscopic finding of normal rat tongue epithelium. (b) Sections of the tongue tissue in rats treated with 4NQO showed a malignant epithelial neoplasm. Tumor cells exhibited unusual characteristics, including the formation of keratin granules; intense lymphocytic infiltration was also observed around the affected areas. The mass was diagnosed as well‐differentiated SCC. (c) The sections of the tongue mass of rats treated with *Lactobacillus plantarum* . The low percentage of keratinocytes at the lower levels of the microscopic slide indicates tissue healing at the higher levels. (d) Tissue sections from the tongue of rats treated with *Lactobacillus casei* . In this slide, we observed a lower percentage of horn cells at lower levels. (Deformed and tumor cells in the tissue are indicated by arrows).

The rats' weight decreased over 16 weeks following exposure to the carcinogenic compound 4NQO. This decrease in weight is illustrated in Figure 3 and was measured weekly from the start of the experiment. No tumors were found to have developed in the control Group 1 rats at the palatal mucosa of the mouth or other mucosal surfaces (Figure 4a). Additionally, no macroscopic changes were observed in the tongues of rats administered 4NQO through week 8. However, from week 10, a whitish tongue thickening was seen, along with a slight loss of definition in the palatal architecture. Moreover, no signs of metastases or swellings of regional lymph nodes were visible on the body surface of rats that had been treated with 4NQO.

Additionally, an increase in tongue size was observed in 4NQO‐administered rats. During carcinogenesis, gross changes such as leukoplakia, erosion, ulceration, and papillary appearance on the dorsum of the posterior tongue appeared. Histopathological findings ranged from hyperplasia (HP) and mild–moderate dysplasia (mmDP) to severe dysplasia (sDP) and in situ carcinoma (ISC), and progressed to well‐differentiated invasive squamous cell carcinoma (SCC). The severity of lesions corresponded to the duration of administration; for example, 50%, 62.5%, and 77.8% incidence rates for tongue cancer were observed in rats treated with 4NQO for 9, 13, and 16 weeks, respectively (Figure 4b). Figure 4c shows the sections of the tongue tissue of rats treated with *LP*. The low percentage of keratinocytes at the lower levels of the microscopic slide indicates tissue healing at the higher levels. Tissue sections from the tongue of rats treated with *LC* demonstrated a lower percentage of horn cells at lower levels (Figure 4d).

### 3.3 Comparison of *DC‐SIGN* Expression With Live and Dead *Lactobacillus* Bacteria

**FIGURE 5:**
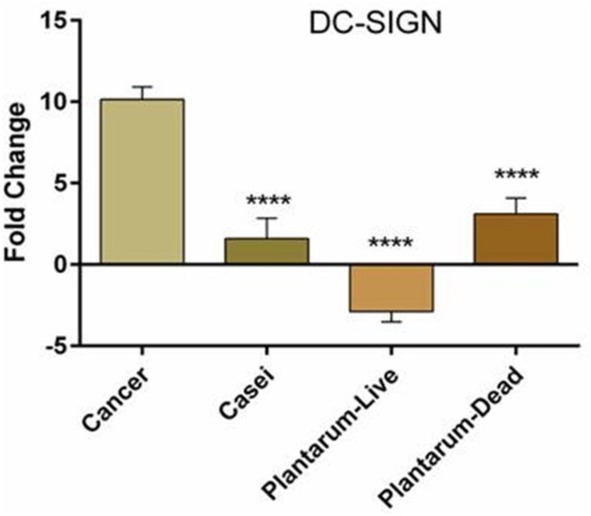
*DC‐SIGN* gene expression in different groups. In the cancer group, *DC‐SIGN* expression increased, and the probiotic bacteria used in this study reduced it in cancer rats. There was no significant difference (ANOVA followed by Tukey post hoc) between live *Lactobacillus casei* and dead *Lactobacillus plantarum* , and both of them decreased the expression of the target gene almost to the same extent. ****p value < 0.0001.

There was no significant difference in *DC‐SIGN* gene expression between the group treated with live *LC* and the group treated with dead *LP*; both reduced the target gene's expression to a similar extent. The expression of this gene in the group treated with live *LP* differed from that in the previous two groups and was placed in a separate group, as shown in Figure 5 (*p* value < 0.0001). Our findings showed that the average expression of *DC‐SIGN* in the *LC* group was 1.249, and its average expression among dead *LP* was 3.295. On the other hand, the mean *DC‐SIGN* expression in the cancer group was 9.945, which differed significantly from that of the previous three groups. In other words, *DC‐SIGN* expression was higher in the cancer group, and probiotic bacteria reduced this expression in cancerous rats (Figure 5).

### 3.4 Comparison of *TLR4* Gene Expression With Live and Dead *Lactobacillus* Bacteria

**FIGURE 6:**
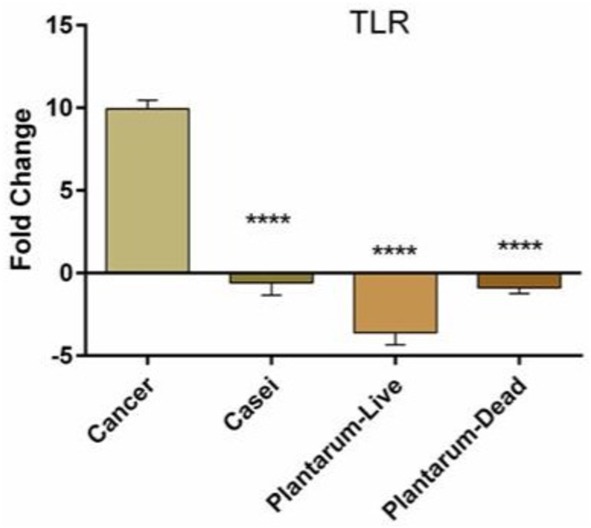
*TLR4* gene expression in different groups. In the cancer group, *TLR4* expression increased, and the probiotic bacteria used in this study decreased its expression in cancer rats. There was no significant difference (ANOVA followed by Tukey post hoc) between live *Lactobacillus casei* and dead *Lactobacillus plantarum* , and both of them decreased the expression of the target gene to almost the same extent. ****p value < 0.0001.

Our study found increased *TLR4* expression at the onset of cancer in rats. However, we observed that administering probiotic bacteria resulted in a significant decrease in this gene's expression in rats (Figure 6). We found no significant difference in *TLR4* gene expression between the groups treated with live *LC* and dead *LP*, and both groups showed a similar degree of decrease. The average relative expression of *TLR4* in the *LC* group was −0.97, and the average relative expression of *TLR4* in the dead *LP* group was −0.731. In contrast, the average relative expression of *TLR4* in the cancer group was 9.884 (*p* value < 0.0001) (Figure 6).

### 3.5 Comparison of *NF‐κB* Gene Expression With Live and Dead *Lactobacillus* Bacteria

**FIGURE 7:**
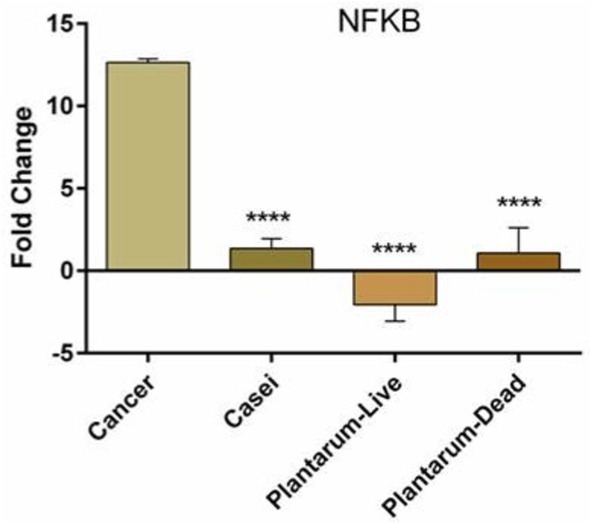
*NF‐κB* gene expression in different groups. In the cancer group, *NF‐κB* expression increased, whereas the probiotic bacteria used in this study decreased it in cancer rats. There was no significant difference (ANOVA followed by Tukey post hoc) between live *Lactobacillus casei* and dead *Lactobacillus plantarum* , and both of them decreased the expression of the target gene almost to the same extent. ****p value < 0.0001.

After rats develop cancer, probiotic bacteria used in this study have been shown to decrease *NF‐κB* gene expression, which is increased in cancerous rats. Based on the data presented in Figure 7, no significant difference in *NF‐κB* gene expression was observed between groups treated with dead *LP* and live *LP*. Additionally, the expression of the target gene was decreased to a similar extent across all three groups, including the group treated with live *LC*. Figure 7 showed that the relative expression of *NF‐κB* in the live *LP* group was −2.635, and the average relative expression of *NF‐κB* in killed *LP* was 0.676, and it was 1.095 in the live *LC* group. In contrast, the average *NF‐κB* expression in the cancer group was 12.483, which differed significantly from that of the other three groups (*p* value < 0.0001).

### 3.6 Comparison of *BCL2* Gene Expression With Live and Dead *Lactobacillus* Bacteria

**FIGURE 8:**
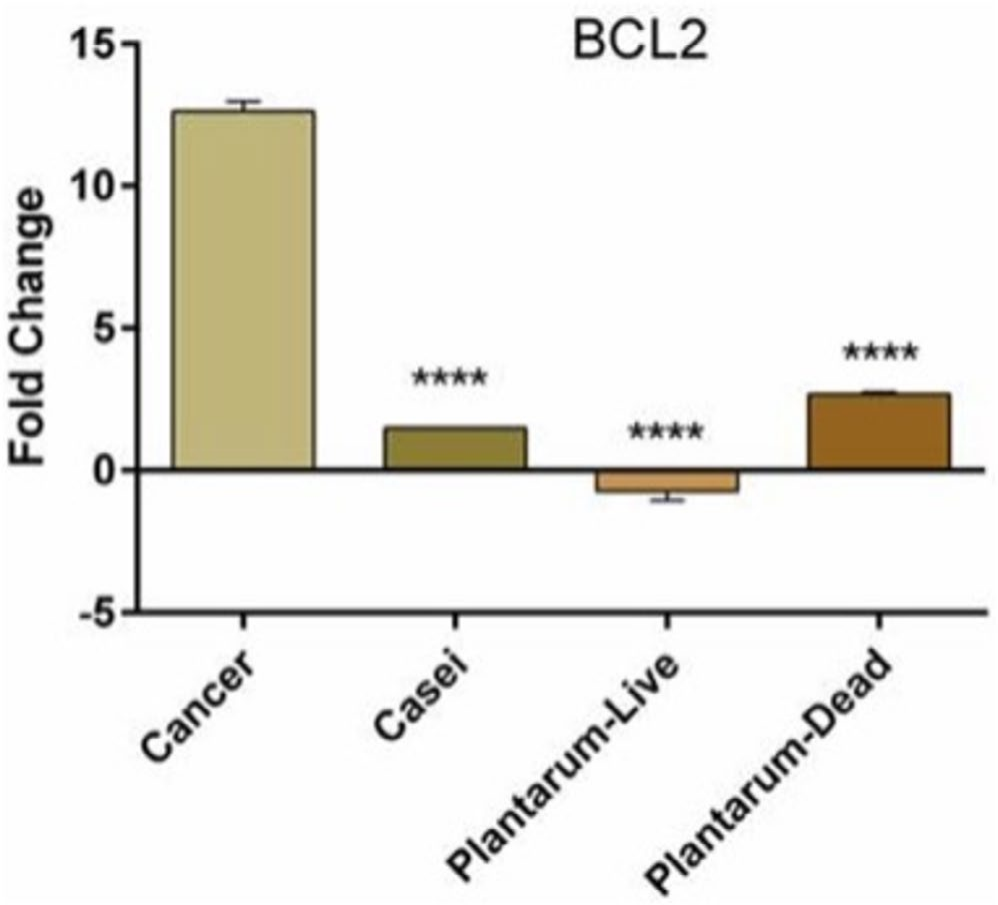
*BCL2* gene expression in different groups. The results indicate increased *BCL2* gene expression in cancerous rats, whereas probiotic bacteria decreased *BCL2* expression. There was a significant difference between the groups, and those treated with live *Lactobacillus plantarum* , live *Lactobacillus casei* , or dead *Lactobacillus plantarum* showed decreased *BCL2* gene expression. ****p value< 0.0001.

The results illustrated in Figure 8 indicate a notable difference between the groups. Specifically, the groups treated with live *LP*, live *LC*, and killed *LC* showed decreased *BCL2* gene expression. The data presented in Figure 8 demonstrated that live *LP* had an average *BCL2* expression of 0.694, live *LC* had an average expression of 1.486, and dead *LP* had an average expression of 2.655. In contrast, the cancer group had a relative *BCL2* expression of 12.595, which differed significantly from those of the other three groups (*p* value < 0.0001). These findings suggest that rats in the cancer group had the highest *BCL2* expression and that treatment with probiotic bacteria resulted in a notable decrease in *BCL2* expression across all treatment groups.

## 4 Discussion

OC is the 16th most common cancer in the world and 1 of the 10 most common cancers in Iran [3, 4]. Recent studies demonstrated that this cancer is related to the combination of oral and intestinal microbiota [11]. Studies conducted on laboratory animals have revealed that probiotic bacteria may reduce the risk of cancer by suppressing tumor growth [31]. While prevention and timely diagnosis are crucial in managing the irreversible complications of cancer, the development of non‐invasive and effective treatment methods that can replace current invasive options (such as surgery, chemotherapy, radiotherapy, etc.) and mitigate their numerous side effects is of utmost significance [35]. OC, being one of the most prevalent gastrointestinal cancers, is among the top 10 causes of death due to its late diagnosis. This is often due to several factors, such as the absence of symptoms in the early stages, the resemblance of clinical features to those of other lesions, and variations in clinical manifestations [36]. With recent progress in cancer treatment, including targeted therapy and immunotherapy, there is renewed optimism for the successful treatment of various types of cancer, including OC [37].

While genetic factors certainly play a role in cancer risk, an individual's immunological status, which is closely linked to probiotic bacteria and typical bacterial flora, particularly in the digestive system, also has a significant impact. The use of specific bacterial properties in the prevention and treatment of cancer has made probiotic strains a promising option [38].

Clinical studies have shown that probiotic consumption can boost natural killer cells (NK cells) activity and immunoglobulin levels. Additionally, Askari et al. showed that *BCL2* expression (2.04%) in patients with esophageal cancer was higher than in normal tissues [39]. Our study confirms that probiotic treatment reduces *BCL2* expression, suggesting increased apoptotic potential in cancer cells.

This study aimed to evaluate the therapeutic properties of *LP* and *LC* as promising probiotic options for cancer treatment in an animal model of OC. Specifically, we aimed to investigate the regulation of downstream signaling pathways of lectin receptors, including the *NF‐κB*, *DC‐SIGN*, *TLR4*, and *BCL2*. In the current study, we showed that *LP* (dead and live) decreased the relative expression of *DC‐SIGN*, *TLR4*, *NF‐κB*, and *BCL2* compared with cancerous rats.

Pasterkamp et al. demonstrated that *DC‐SIGN* can initiate innate immune responses against mast cells by modulating *TLR* signaling [40]. *TLRs* are expressed by various cell types, including antigen‐presenting cells (such as dendritic cells and macrophages), fibroblasts, and epithelial cells [26]. However, Ridnour et al. showed that regarding the relationship between *TLR* expression and cancer prognosis. For instance, the same receptor can be linked to either good or poor outcomes in different cancers (such as *TLR9*) or generally associated with unfavorable outcomes (such as *TLR4*) [41]. This complexity highlights the challenges of studying *TLRs* in oncogenesis and cancer progression and suggests that investigating a single receptor in different cancer types may not be the most effective approach. Our study showed that *DC‐SIGN* expression was significantly increased in the cancer group but was notably reduced following probiotic treatment. Also, *TLR4* expression was significantly upregulated in OSCC rats, indicating its role in promoting tumor progression and inflammation. However, we also provide new evidence that probiotic treatment significantly downregulated *TLR4* expression, suggesting that targeting *TLR4* through Probiotic intervention may help suppress tumor‐related inflammation and immune evasion mechanisms.

*TLRs* are then activated and recruit other proteins within immune cells, thereby propagating the antigen‐induced signaling pathway. The recruited proteins activate downstream proteins, including protein kinases, which amplify the signal and ultimately lead to the upregulation or suppression of genes that regulate inflammatory responses and gene transcription [42]. These processes affect different cell populations, such as cancer stem cells, cancer cells, tumor‐infiltrating lymphocytes, and tumor‐associated fibroblasts, which may exhibit varying *TLR* expression and responses to *TLR* stimulation [43]. The decrease in *DC‐SIGN* expression observed in mice treated with lactobacilli in this study could potentially improve cancer prognosis and decrease invasion. Lactobacilli affect *only TLR4*, and the reduction in *DC‐SIGN* expression is accompanied by a reduction in *TLR4* expression, which may lead to decreased activation of cancer cells.

We also assessed the impact of live and dead *LP* and live *LC* treatments on *TLR4* gene expression in cancerous rats compared to the control group. The findings revealed a significant difference between the cancerous group and the three groups treated with bacteria. The expression of *TLR4* was lower in the bacterial treatment groups than in the cancerous group, with a greater decrease observed in the live *LP*‐treated group. Therefore, it can be inferred that live *LP* was more effective in reducing *TLR4* gene expression. Xia et al.'s study, in accordance with our study, found that *LP* and *LC* were two to three times more effective than other strains in reducing epithelial damage indices and improving colon health (by 10%–30%) while maintaining the integrity of the epithelial barrier in rats with colitis. The study also revealed that the anti‐inflammatory effect of probiotics is associated with increased hemoxygenase‐1 (HO‐1) expression and decreased expression of the *TLR4/MyD88/NF‐kB* pathway in mouse colon tissues. Furthermore, they showed that supplementation with *LP* partially reduces the dysbiosis of the intestinal microbiota caused by DSS (Sodium trimethylsilylpropanesulfonate) treatment [44]. Liu et al. found that *LP* was able to regulate the expression of genes related to lipid transfer and activation of the *TLR4/NF‐κB* signaling pathway, which was in accordance with our findings [45]. Elshaer et al. highlighted the role of *LP* in preventing liver cirrhosis through suppression of *TLR4/CXCL9/PREX‐2* and found that, in line with our study, early administration of *LP* could significantly reduce the expression of *TLR4*, *CXCL9*, and *PREX‐2*, along with the improvement of liver function and preventing pathological changes [46]. However, it is important to note that *TLR4* is not only expressed in cancer cells but also in immune cells, as evaluated in this study. Therefore, this marker can have a dual effect, acting as a double‐edged sword. While its activation by lactobacilli in cancer cells may reduce cancer progression, its simultaneous activation in immune cells may increase inflammation and have contradictory effects on the response to cancer cells. To fully understand these contradictions, it is recommended to evaluate them in a wider study.

In the current study, *NF‐κB* gene expression was evaluated in both the control and cancerous groups treated with live and dead *LP* and *LC*. The results revealed a significant difference between the group treated with live *LC* and dead *LP* and the cancerous control group. The decrease in *NF‐κB* gene expression was more pronounced in the group treated with live *LP*. These results are consistent with the findings of Huang et al.'s study [47], which also reported an increase in *NF‐κB* expression in cancer cells and a significant decrease in *NF‐κB* expression after treatment with lactobacilli. Yue et al. conducted a study on the impact of *LP* and its cell‐free supernatant on colon cancer in mice. The results showed that this treatment prevented the development of colon tumors and mucosal damage by modulating the immune system, reducing the expression of inflammatory cytokines (*IL‐6, IL‐17F*, and *IL‐22*), and limiting the infiltration of inflammatory cells. Furthermore, *LP* inhibited *NF‐κB* and Wnt signaling pathway activation while restoring gut microbiota composition to levels similar to those of wild‐type mice [48]. Qing et al. discovered that *LP* can prevent the development of Aflatoxin B1 (*AFB1*) and chronic liver damage caused by mycotoxins. This is achieved through the modulation of *TLR2/NF‐κB* and *TLR4/NF‐κB* pathways, which results in the secretion of anti‐inflammatory cytokines [49].

In this study, the expression of the *BCL2* gene was evaluated in control and patient groups treated with live and dead *LP* and *LC*. The cancer group showed the highest *BCL2* gene expression, whereas treatment with probiotic bacteria resulted in a significant decrease in *BCL2* gene expression across all treatment groups [50]. A study by Sentürk et al. evaluated the toxicity of secondary metabolites of *LP* on the human breast cancer cell line (*MCF‐7*) and *Drosophila melanogaster* . The study found that the expression levels of anti‐apoptotic genes *BCL2* and *BUFFY* (*BCL2‐48AE*) were decreased in both species, while expression of the apoptotic genes *DECAY* (Death executioner caspase related to Apopain/Yama), *FADD* (Fas associated via death domain), and *RAS64B* (Ras oncogene at 64B) were increased in Drosophila [51]. Kim et al. demonstrated that extracts of *LP* inhibited *Staphylococcus aureus* ‐induced cell death in the human epithelial cell line *HT‐29* [52].

Our study on the effects of *LC* on an OSCC rat model found a significant difference between the live *LC* treatment group and the cancerous control group. Specifically, the average expression of the *DC‐SIGN* gene in the *LC* treatment group was 1.249, which was significantly lower than that in the cancer group (9.945). Soltan Dallal et al. demonstrated the effectiveness of *LC* in preventing the growth of graft tumors in experimental animal models [53]. We also showed that the average expression of the *TLR4* gene in the group treated with *LC* was 0.97, which was significantly different from the cancer group with an average of 9.884. A study by Chung et al., which aligned with our findings, evaluated the effects of *LC* on colon‐related microbiota, mucosal cytokine balance, and *TLR* expression. The study found that rectal administration of *LC* significantly decreased the mRNA levels of *TLR‐4* and *IL‐1β*, while significantly increasing mucosal IL‐10 levels [54]. A study conducted by Watanabe et al. investigated the effect of *LC* on small intestine damage caused by indomethacin. In contrast to the current research, their findings showed that a one‐week treatment with live *LC* increased lactic acid concentration in the small intestine, inhibited the increase in myeloperoxidase activity, and reduced *TNF‐α* mRNA expression. However, the treatment did not affect *TLR4*. It is important to remember that activating the *TLR4* pathway can trigger an inflammatory response that enhances the tumorigenic potential of cancer cells and helps them evade the immune system. As a result, the decrease in *TLR4* expression observed in the groups treated with specific lactobacilli in this study, compared with OC cells, may be advantageous for reducing *TLR4*‐dependent inflammation and ultimately lowering the cancer cells' tumorigenic potential.

According to the current study's findings, the average *NF‐κB* expression in the *LC‐tr*eated group was 1.095, which was significantly lower than that in the cancer group (12.483). These results are consistent with Liu et al.'s study, which reported inhibitory or modulatory effects of lactobacilli on *NF‐κB*‐related pathways, resulting in reduced expression of this gene [45]. Tilborghs et al. showed that the *NF‐κB* signaling pathway is continuously activated in many malignancies, particularly in gastrointestinal cancers, resulting in abnormal cell proliferation and differentiation, increased metastasis, and treatment resistance [55]. The present study demonstrated that treatment with lactobacilli led to a decrease in the expression of *NF‐κB* compared to cancer tissues. As a result, it is expected that the tumor's cell proliferation, survival, angiogenesis, inflammation, expansion, and metastasis will be reduced. However, further studies are needed to evaluate these hypotheses in more detail.

The current study also found that the average *BCL2* expression in the *LC*‐treated group was 1.486, which was significantly lower than the cancer group's average of 12.595. The overexpression of *BCL2* in cancer cells may inhibit pro‐apoptotic signals, allowing cancer cells to survive under stressful conditions. Kang et al. reported that High levels of pro‐apoptotic proteins, which are then cleared by increased *BCL2* expression, may lead to a phenomenon called the primary state [56]. The present study showed that the expression of *BCL2* in the group treated with lactobacilli was lower than in the cancer group, suggesting that the decrease in expression may be associated with reduced cell proliferation, survival, angiogenesis, inflammation, tumor expansion, and metastasis. However, further studies are needed to evaluate these assumptions in more detail.

We also compared the performance of live and dead *LP* with that of *LC*. These results indicated that cancer cells exposed to live *LP*, dead *LP*, or *LC* exhibited decreased expression of *TLR, DC‐SIGN, BCL2*, and *NF‐κB* genes compared with the cancer control group. However, a comparison of gene expression among the groups revealed that live *LP* was more effective against cancer cells than either dead *LP* or *live LC*. Additionally, the expression of these genes in live *LP* and *LC* decreased at similar rates, suggesting that these two bacteria exhibit comparable anticancer properties. Therefore, we conclude that live *LP* was the most potent bacterium against cancer cells among the tested strains.

## 5 Conclusion

In this study, an animal model of OC induced by 4NQO was used to investigate the potential therapeutic effects of *LP* and *LC* probiotics under normal immune‐response conditions. After confirming the model, the effects of probiotics on the *DC‐SIGN*, *TLR4*, *NF‐κB*, and *BCL2* genes were evaluated. Although there was no significant difference among the three groups treated with live *LC* and live or dead *LP*, all treatments showed significantly lower expression of these genes than in the cancer group. This reduction in gene expression suggests decreased chronic inflammation and reduced production of inflammatory factors and cytokines, while increasing the likelihood of inducing apoptosis in cancer cells. Overall, the use of probiotics, particularly lactobacilli, can positively affect downstream signaling pathways, such as *NF‐κB*.

## 6 Limitations and Strengths

This study demonstrates the potential of *LP* and *LC* for OSCC treatment by modulating immune pathways, including *NF‐κB, TLR4*, and *DC‐SIGN*. The use of an OSCC rat model enhances its relevance, while molecular and histopathological analyses provide a comprehensive evaluation of probiotic effects. Comparing live and killed probiotics offers insights into bacterial viability in cancer modulation. Ethical compliance ensures research credibility, reinforcing probiotics as a promising therapeutic approach. However, the study has limitations. It lacks long‐term tumor progression analysis to assess the sustained impact of probiotics on tumor growth and metastasis. A detailed immunological assessment, including cytokine profiling, would enhance mechanistic insights. The absence of validation in human OSCC cell lines limits translational applicability. Additionally, variations in probiotic dosage and microbiome composition were not explored, restricting a full understanding of their effects. Addressing these gaps through dose–response studies and microbiome analysis is essential to clarify the therapeutic potential of probiotics in OSCC treatment.

## Author Contributions

**Vahideh Faghanizadeh:** investigation, writing – original draft, writing – review and editing, formal analysis, data curation. **Malihe Naderi:** writing – original draft, methodology, writing – review and editing, formal analysis. **Nazila Arbab Soleimani:** conceptualization, investigation, funding acquisition, writing – original draft, writing – review and editing, visualization, validation, methodology, software, formal analysis, project administration, resources, data curation, supervision. **Ayyoob Khosravi:** conceptualization, methodology, project administration, supervision, writing – original draft, writing – review and editing. **Mohammad Mahdi Forghanifard:** writing – original draft, writing – review and editing, methodology, data curation, software.

## Funding

This study did not receive any external funding. All expenses were covered personally by the authors.

## Ethics Statement

We confirm that the animal study titled has been approved by the Ethics Committee on Animal Research of Islamic Azad University, Damghan, Iran, under the approval number 1401.002. All efforts were made to minimize the animals' discomfort, distress, and pain, and the study was conducted in accordance with the highest ethical and scientific standards.

## Conflicts of Interest

The authors declare no conflicts of interest.

## Data Availability

The data that support the findings of this study are available from the corresponding author upon reasonable request.
